# Draft Genome Sequence of *Vibrio fortis* Dalian14 Isolated from Diseased Sea Urchin (*Strongylocentrotus intermedius*)

**DOI:** 10.1128/genomeA.00409-14

**Published:** 2014-07-03

**Authors:** Jun Ding, Yan Dou, Yinan Wang, Yaqing Chang

**Affiliations:** Key Laboratory of Mariculture & Stock Enhancement in North China’s Sea, Ministry of Agriculture, Dalian Ocean University, Dalian, China

## Abstract

Here, we report the draft genome sequence of *Vibrio fortis* Dalian14 isolated from diseased sea urchin (*Strongylocentrotus intermedius*) during disease outbreaks in North China. The availability of this genome sequence will facilitate the study of the mechanisms of pathogenicity and evolution of *Vibrio* species.

## GENOME ANNOUNCEMENT

*Vibrio* species are ubiquitous in the marine environment and are implicated as the causes of several diseases in wild and cultured aquatic organisms, such as farm shrimps and sea urchins (1–3). Bacterial diseases involving sea urchins have been reported in the wild as well as in aquacultures and laboratory aquariums. The bacteria involved in the mortality of sea urchins include *Shewanella*, *Pseudoalteromonas*, and *Vibrio* species (2). *Vibrio fortis* Dalian14 was isolated from diseased sea urchins (*Strongylocentrotus intermedius*) from the Heishijiao hatchery of Dalian Ocean University in Dalian during disease outbreaks. *V. fortis* Dalian14 was found to be sensitive to ampicillin, enrofloxacin, ofloxacin, doxycycline, and florfenicol.

Genome sequencing was performed with the Illumina HiSeq 2000 platform. A genomic library with a 300-bp insert size was constructed and sequenced, providing about 180-fold coverage of the genome. *De novo* assembly was performed using SOAP*denovo*2 (4). BLASTn (5) similarity searches were conducted against the bacterial protein database (ftp://ftp.ncbi.nlm.nih.gov/genomes/Bacteria) with the scaffolds, and the best matched genome was selected as the reference genome. Next, we performed LASTZ and Chain/Net (6) to order the scaffolds. The gaps within and between the scaffolds were closed with GapFiller (7). The open reading frames were identified using Glimmer version 3.02 (8). The tRNAs and rRNAs were predicted using tRNAscan-SE (9) and RNAmmer (10), respectively. The functions of encoding genes were annotated using the NCBI nr, Swiss-Prot (11), Clusters of Orthologous Groups (COG) (12), KEGG (13), and InterProScan (14) databases.

The draft genome sequence consists of 33 scaffolds and 35 contigs, with a mean G+C content of 44.92% and a total length of 5,286,006 bases. A total of 4,558 coding sequences (CDSs) were predicted. Approximately 78.93% of all coding sequences (a total of 3,598) were assigned to COGs, and the CDSs can be annotated into the 165 pathways using KAAS (15).

### Nucleotide sequence accession number.

This whole-genome shotgun project has been deposited at DDBJ/EMBL/GenBank under the accession no. JFFR00000000.
